# Histopathology reveals environmental stress in dusky flounder *Syacium papillosum* of the Yucatan Peninsula continental shelf

**DOI:** 10.1007/s10661-024-12996-2

**Published:** 2024-09-06

**Authors:** Eunice Danilú Couoh-Puga, María Cristina Chávez-Sánchez, Víctor Manuel Vidal-Martínez, Gerardo Gold-Bouchot, Oscar Arturo Centeno-Chalé, M.Leopoldina Aguirre-Macedo

**Affiliations:** 1 Centro de Investigación y de Estudios Avanzados del Instituto Politécnico Nacional/Unidad Mérida, Departamento de Recursos del Mar, Km 6 Carretera Antigua a Progreso, Cordemex, Mérida, 97319 México; 2 Centro de Investigación en Alimentación y Desarrollo en Acuicultura y Manejo Ambiental (CIAD), Av. Sábalo-Cerritos, Mazatlán, Sinaloa, 82112 México; 3 Facultad de Química, Unidad de Química en Sisal, Universidad Nacional Autónoma de México, Sisal, Yucatán México; 4 Oceanography Department and Geochemical and Environmental Research Group, Texas A&M University, College Station, TX USA

**Keywords:** Yucatan shelf, Anthropogenic pollution, Histological changes, Flatfish, Histological index

## Abstract

The histological changes in the liver, kidney, spleen, and gills of *Syacium papillosum* from the continental shelf of the Yucatan Peninsula, Gulf of Mexico, and their statistical associations with environmental conditions and pollutants were assessed in 2010, 2011, and 2012. We evaluated the extension and severity of the lesions through a degree of tissue change (DTC), and, with the sum of the number of lesion types within each of their DTC stages, we determined the histological alteration index (HAI). The liver and kidney were the most affected organs, with HAI values > 100. Fish with the most severe damage were observed on the Campeche Bank and the Caribbean Sea, contrasting with those collected from the northern Yucatan continental shelf. The presence of foci cellular alteration and abundant melanomacrophage centers indicated that these flatfishes were chronically exposed to environmental stress factors. Redundancy analyses showed strong associations between HAI values and hydrocarbon and heavy metal concentrations in muscle. Our results provide evidence for the first time of a differential health condition of the Yucatan shelf through the histopathology shown in *S. papillosum*, establishing the baseline for future monitoring programs in the region.

**Graphical abstract:**

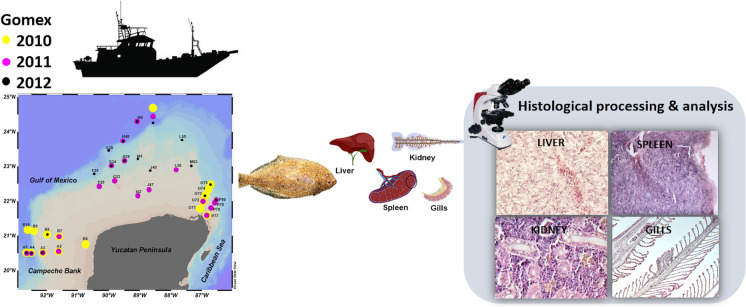

**Supplementary Information:**

The online version contains supplementary material available at 10.1007/s10661-024-12996-2.

## Introduction

The Gulf of Mexico (GoM) is an important source of marine ecosystems; and the anthropogenic activities developed there have produced chronic inputs of contaminants, such as heavy metals and hydrocarbons (Gold-Bouchot et al., 2023) . The oil extraction and transportation processes in shallow and deep waters throughout the GoM often have resulted in spills of various magnitudes (Pulster et al., 2020). The release of chemical oil products due to intense maritime transportation also represents an important pollution source that can carry out catastrophic effects on biota (Hook et al., 2014; Vidal-Martínez et al., 2019). Additionally, high concentrations of metals, hydrocarbons, and other chemical contaminants from the environment can be accumulated by marine organisms (Bazzi, 2014; Quintanilla-Mena et al., 2019).

Fish are sensitive to toxicants and considered as potential indicators of environmental pollution. Consequently, fish have been proposed as “effect indicators” and “accumulation indicators” because of the variety of ways in which they respond to pollutants such as oil spills, heavy metals, domestic sewage, and agricultural and industrial contamination (Authman, 2015; Tashla et al., 2018). Toxic compounds can be absorbed into the organism’s organs, tissues, and cells, resulting in different degrees of histological lesions, immune responses, and in many cases, harmful diseases (Améndola-Pimenta et al., 2020). In this sense, pathological fish histology can be a helpful tool for evaluating and monitoring aquatic ecosystems (Couoh-Puga et al., 2021; Yancheva et al., 2016). An advantage of histopathological responses is their integrative potential because they reflect an intermediate effect between the biochemical and individual responses. Moreover, different tissue structural changes are considered a response to several environmental threats, thus reflecting the impact of the total environmental mixture of toxic compounds in the respective area (Teh et al., 1997; Gusso-Choueri et al., 2022). Recently, Murawski et al. (2014) and Ali et al. (2014) reported skin lesions in red snapper (*Lutjanus campechanus*) and splenic responses in sea trout individuals exposed to different hydrocarbons, such as polycyclic aromatic hydrocarbons (PAHs) from the Deepwater Horizon (DWH) oil spill. Despite unexposed trout fish developing sporadic melanomacrophage centers (MMCs), those were significantly greater in number and size in the fish exposed in the GoM.

Histological lesions caused by environmental pollution have been well documented (Khoshnood, 2016; Yancheva et al., 2016). Liver and gill lesions in marine fish, for example, neoplasms, non-neoplastic proliferative lesions, and specific degenerative/necrotic lesions, have been associated with exposure to chemical xenobiotics and are well-established as histological bioindicators in response to pollutants (Adams, 2003; Rojo-Nieto et al., 2014). Moreover, the presence of liver tumors in bottom-dwelling fish has been clearly associated with PAHs in sediment. However, exposure to other chemicals can likely contribute either as an initiator or as a promoter (USEPA et al., 2012). As a response, fish health may reflect a low nutritional status and susceptibility to chemical exposure (Schmitt & Dethloff, 2000). Subsequently, physiological processes can be affected, causing fish death in severe cases (Vázquez-Gómez et al., 2016). In this sense, the physiological responses in fish fitness (estimated as the condition factor (*K*)) also can be affected. Such responses have been observed as an inverse relationship between the condition factor *K* and histopathological changes of fishes in environmentally polluted conditions (Javed & Usmani, 2019).

In this context, one of the most significant threats in in the Gulf of Mexico (GoM) in recent years has been the DWH oil spill, which occurred in April 2010. The release of approximately 134 million gallons (4.9 million barrels) of oil at 1600 m depth into the GoM required the addition of 1.84 million gallons of dispersant (Pallardy, 2023; Soto & Botello, 2013), the ecological impact continue under investigation. Although this oil spill was located within 555 km of the Yucatan shelf (YS) (CONAGUA, 2014), there was still concern that its effects could reach the YS. However, the organisms living in the YS could be affected by other environmental problems not yet evaluated, such as pollutants arrived from Cayo Arcas offshore crude oil loading terminal or the heavy maritime traffic of ships that transport petroleum coming from Aruba, Curacao, Maracaibo, and Trinidad and Tobago, all of which use the Yucatan Channel as a frequent route of transportation to North America (Botello, 2005). In addition, tourism activities from the Caribbean zone and the presence of natural hydrocarbon seeps could also contribute to YS pollution (Botello, 2005; Love et al., 2013).

The DWH oil spill established the importance of distinguishing local YS environmental damage sources from their external counterparts. Thus, an environmental baseline of the YS had to be established. This accident gave us an opportunity to start the GoM monitoring program. The National Institute of Ecology and Climatic Change Center (I.N.E.C.C.), together with other national marine and oceanological institutions, implemented a monitoring plan to establish baseline environmental conditions in the southern Gulf of Mexico after the DWH catastrophe. As a component of such baseline establishment, this paper aims to record flatfish (*Syacium papillosum*) histopathological alterations that may indicate the presence and effects of pollutants on the Yucatan shelf. We hypothesized that severe histological lesions would be present in flatfishes of the sampling sites closest to the oil spill–impacted area and that histological alteration index scores (HAI) would be statistically associated with environmental and contamination variables in sediments and organisms.

Histological biomarkers provide advantages over other environmental stress biomarkers; first, they provide identification of specific target organs and tissues during exposure to potentially toxic pollutants; moreover, they can be used to establish the specific patterns of both acute and chronic deleterious effects of such pollutants on tissues (Au, 2004).

Therefore, the scarce information on the study area about temporal dynamics of xenobiotic exposure in the area after the DWH oil spill and the tissues morphological changes of *S. papillosum* as a potential bioindicator contributes to the originality and value of this research as future monitoring programs in YS.

Thus, the study aims were (i) to establish quantitative values through a histological index, (ii) to determine whether the histological changes in *S. papillosum* exhibit differences among subregions from east to west of the YS, and (iii) to determine whether the occurrence of histological damage of *S. papillosum* is associated with natural physicochemical environmental variables, heavy metals, nutrients, and/or hydrocarbons at the seascape level.

## Materials and methods

### Study sites and fish sampling

**Fig. 1:**
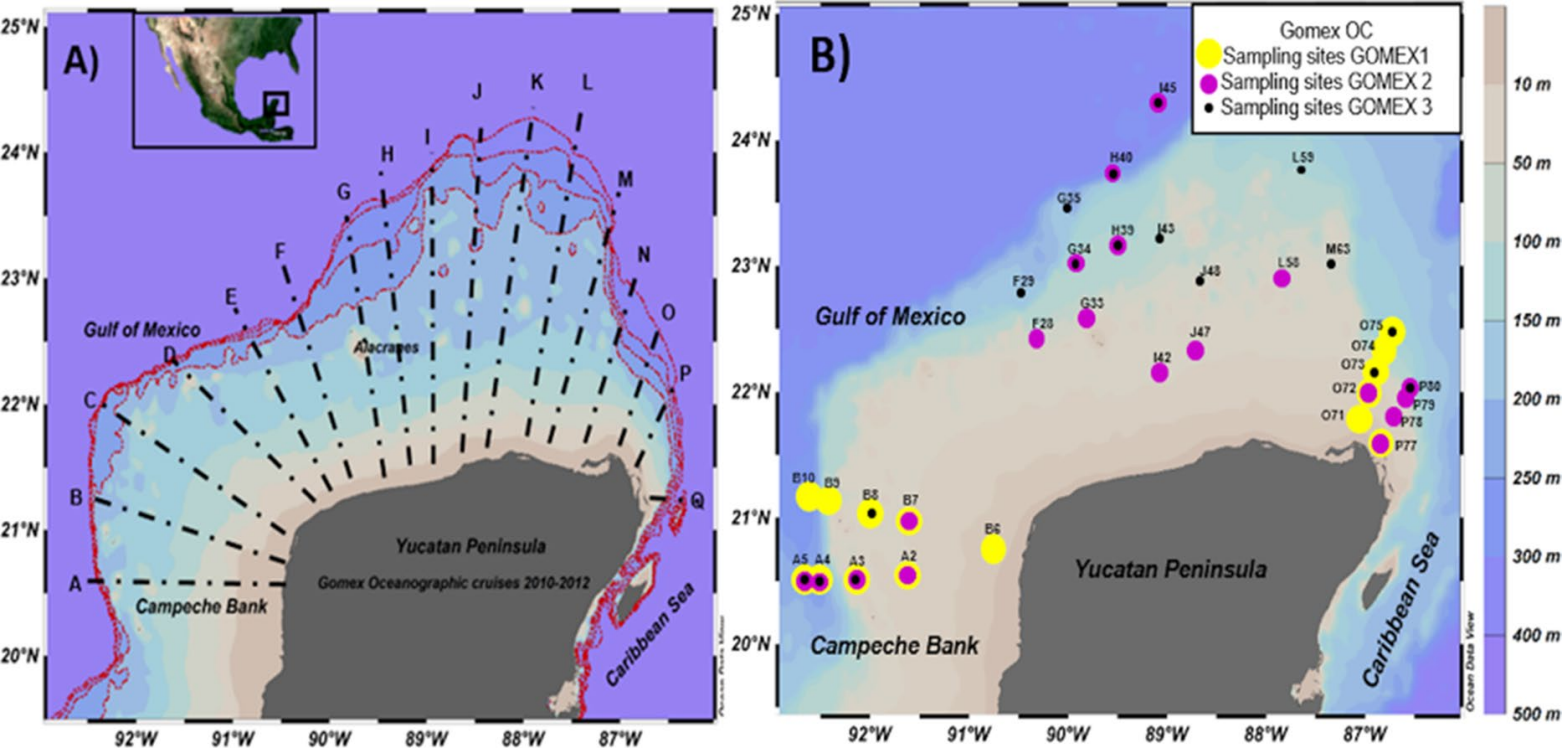
Study area. **A** Sampling transect network for the Gomex oceanographic cruises (GMX) over the Yucatan continental shelf during 2010–2012, A–E transects of the region near the Campeche Bank (Campeche Bank zone); F–K transects of the central zone of the Yucatán shelf (Yucatán zone); L–Q transects of the Mexican Caribbean (Yucatan-Quintana Roo zone). **B** Sampling sites where fish were collected during the Gomex oceanographic cruises 2010–2012

The study area comprised 87 sampling sites on the continental shelf of the Yucatan Peninsula from Isla Arena, Campeche, in the west to Playa del Carmen, Quintana Roo, in the east (Fig. 1). The sampling sites covered the northern shelf of the entire peninsula between 15 and 200 m depth.

Three oceanographic cruises (OC) were carried out during consecutive years: Gomex-1 (10–21 September 2010) and Gomex-2 (September 23 to October 3, 2011), during the rainy season, and Gomex-3 (from November 28 to December 2012) during the early north winds season. Once in sampling sites, the fish were collected by net trawling and identified by ichthyologists of the Necton Laboratory (CINVESTAV-IPN, Mérida).

In each OC, a total of 62 environmental variables from water and sediments were measured (hydrocarbons and heavy metals), including bile PAH metabolites in organisms (Supplementary material Table S1). In addition, nutrients and physicochemical water parameters (e.g., oxygen (mg/l), salinity (psu), and temperature (°C)) were measured (Supplementary material Table S1). The physicochemical characteristics and concentrations of hydrocarbons and heavy metals in sediments and organisms were determined at the Marine Geochemistry Laboratory (CINVESTAV-IPN, Mérida) applying standardized and recommended methods (Botello et al., 2015). PAH metabolite concentrations in organisms were provided by the Ecotoxicology Laboratory following standardized methods (Gold-Bouchot et al., 2017).

Immediately after being caught, fish morphometric data were taken. The fish were weighed (g) and measured for total length (cm) and then dissected. The length–weight relationship (LWR) of *S. papillosum* was estimated using the equation *W* = *α*SL^β^ (Ricker, 1975), where *W* = total weight (g), SL = standard length (cm), *α* is the ordinate to the origin, and *β* is the intercept, also known as the allometry coefficient. Based on the estimations of LWR, we used the condition factor (*K*) proposed by Bagenal and Tesch (1978) (*K* = 100 × *W*/SL^β^) to assess the physiological condition of the fish, as well as the effect of pollutant exposure on this physiological biomarker (Couoh-Puga et al., 2021; Snyder et al., 2019).

### Histopathological analyses

Each fish was euthanized by brain puncture, to avoid mechanical damage to tissues, mainly in gills, previously reported by anesthetics (Boijink et al., 2017; Couoh-Puga et al., 2021). All organisms were handled under the Guidelines for Care and Manipulation of Laboratory Animals of Cinvestav, the Mexican Official norm NOM-062-ZOO-1999 (http://www.fmvz.unam.mx/fmvz/principal/archivos/062ZOO.PDF) and the Guide for the Care and Use of Laboratory Animals as adapted by the US National Institutes of Health (http://www.aaalac.org/resources/Guide_2011.pdf). Gills, kidney, liver, and spleen tissue sections were fixed immediately in a 10% buffered formalin solution to preserve biological tissues and avoid autolysis or putrefaction.

The tissues were processed following the methodology by Humason (1962) and Luna (1968), dehydrated in an automatic processor (Histokinette), and embedded in paraffin (melting point 56 °C). Afterwards, sections were cut to 5-µm thickness using a microtome and mounted in Entellan® for microscopy. Finally, the slides were stained with hematoxylin and eosin (H&E) for morphological examination under an Olympus BX50 compound microscope (Chávez-Sánchez et al., 2014). Using a Q imagine digital camera of 5-megapixel resolution, photographs of normal and histopathological tissues were taken for the histological baseline comparison. All the material corresponding to this investigation is available for consultation in the Aquatic Pathology Laboratory Histology Section of CINVESTAV-IPN, Mérida.

Histological alterations in tissues and organs were evaluated semi-quantitatively based on two criteria according to Schwaiger et al. (1997) and Simonato et al. (2008): (a) extent of the damage (EOD), where the alterations were classified into three degrees of incidence, and (b) degree of tissue change (DTC) based on the severity of the lesions and the possibility of recovery of tissue and organs (Table 1).

**Table 1:** Evaluation of the histopathological alterations in relation to the extent of the damage (EOD) and the degree of tissue change (DTC) or severity of damage

EOD	The extent of the damage	DTC	Histopathological alterations
Grade 1	There are no focal alterations	Stage I	Changes that do not damage the organ to such an extent that it cannot repair itself if conditions improve
Grade 2	Focal changes	Stage II	Changes that are more severe and affect the associated organ or tissue function
Grade 3	Extensive damage	Stage III	Changes that prevent the organ from repairing itself even if conditions improve

The histological alteration index (HAI) was calculated with the equation used by Silva and Martinez (2007) and Simonato et al. (2008) and proposed by Poleksic and Mitrovic-Tutundzic (1994).

$$HAI= 1\cdot \sum I+10\cdot \sum II+100\cdot \sum III$$

In this equation, I, II, and III are the histological lesion stages (DTCs). The HAI was calculated for each fish and tissue. The average HAI was ranked into five categories: 0–10, normal organ; 11–20, slightly to moderately damaged organ; 21–50, moderately to heavily damaged organ; 51–100, severely damaged organ; and > 100, irreversibly damaged organ (Améndola-Pimenta et al., 2020; Chávez-Sánchez et al., 2014). The histopathological alterations were compared among the fish of sampling sites of the same cruise and afterward compared among the fish HAI data on different oceanographic cruises.

### Data analyses

Normality of both EOD mean values and HAI scores was obtained for each organ for each oceanographic cruise and then assessed with Shapiro–Wilks normality test. If normality was not reached, a nonparametric ANOVA test (Kruskal–Wallis) with a level of significance of *p* < 0.05 was used to determine potential differences in HAI values among subregions (see below).

Nonmetric multidimensional scaling (NMDS) was performed with all the HAI values of the four organs examined for the fish collected at every sampling site, considering the depth and zone as grouping factors. For these analyses, we used Primer 6 software, based on the Bray–Curtis dissimilarity index, to determine the patterns that are formed concerning HAI with respect to depth (10–49 m, 50–100 m, 101–200 m) and zone (Campeche Bank, Central Yucatan, Caribbean) factors within the YS and for three Gomex cruises from 2010 to 2012. Due to their geographical proximity, all of the individual fish obtained were grouped a priori into subregions: Campeche Bank in the western YS (sampling sites A2, A3, A4, A5, and B6, B7, B8, B9, B10), Central Yucatan (sampling sites F28, F29, G33, G34, G35, H39, H40, I42, I43, I45, and J47, J48), and the Caribbean (sampling sites L58, L59, M63, O71, O72, O73, O74, O75, P77, P78, P79, and P80). Redundancy analyses (RDA) were performed between dependent variables (HAI values) and independent variables (62 variables: physicochemical, nutrient, and contaminant data for sediments and organisms) (Supplementary material Table S1). Monte Carlo permutations were used to determine the significance of the canonical axes based on 4999 permutations. The independent variables were selected by forward stepwise analyses, keeping only the variables that explained the greatest variance (10%) and did not present a variance inflation factor (*VIF*) higher than four. The significance of the statistics was determined with *p* < 0.05. RDAs were performed with CANOCO software for Windows 5.0 (Ter Braak & Smilauer, 2012), to these analyses, length, weight, and *K* were considered covariables.

## Results

### Water quality

**Fig. 2:**
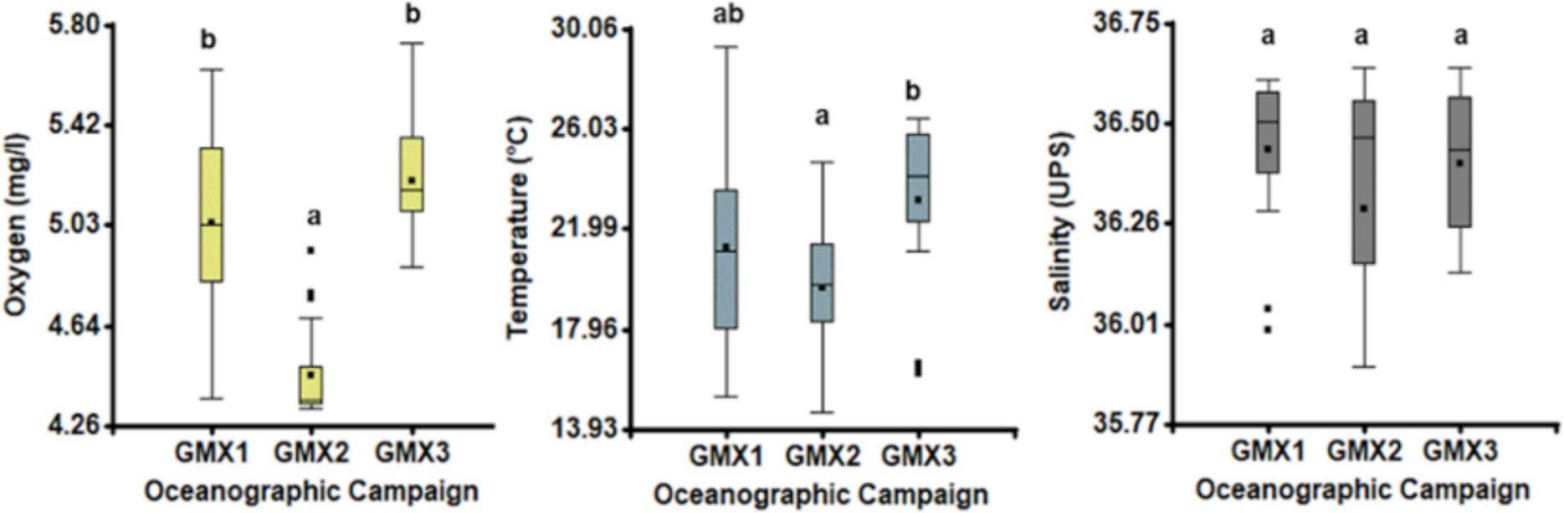
Statistical differences in the oxygen and temperature during three Gomex (GMX) oceanographic cruises (OCs). (A) Mean oxygen ± S.D., (B) mean temperature ± S.D., and (C) mean salinity ± S.D. Different letters denote significant differences (*p* < 0.05). We used Kruskal–Wallis nonparametric one-way ANOVA

The physicochemical parameters of water and pollutants in sediments and organisms are presented in the Supplemental material (Table S1). Analysis results showed that except for salinity, the physicochemical parameters of water (i.e., oxygen, temperature) were variable concerning to the three OCs (Fig. 2).

### Fish sampled

**Fig. 3:**
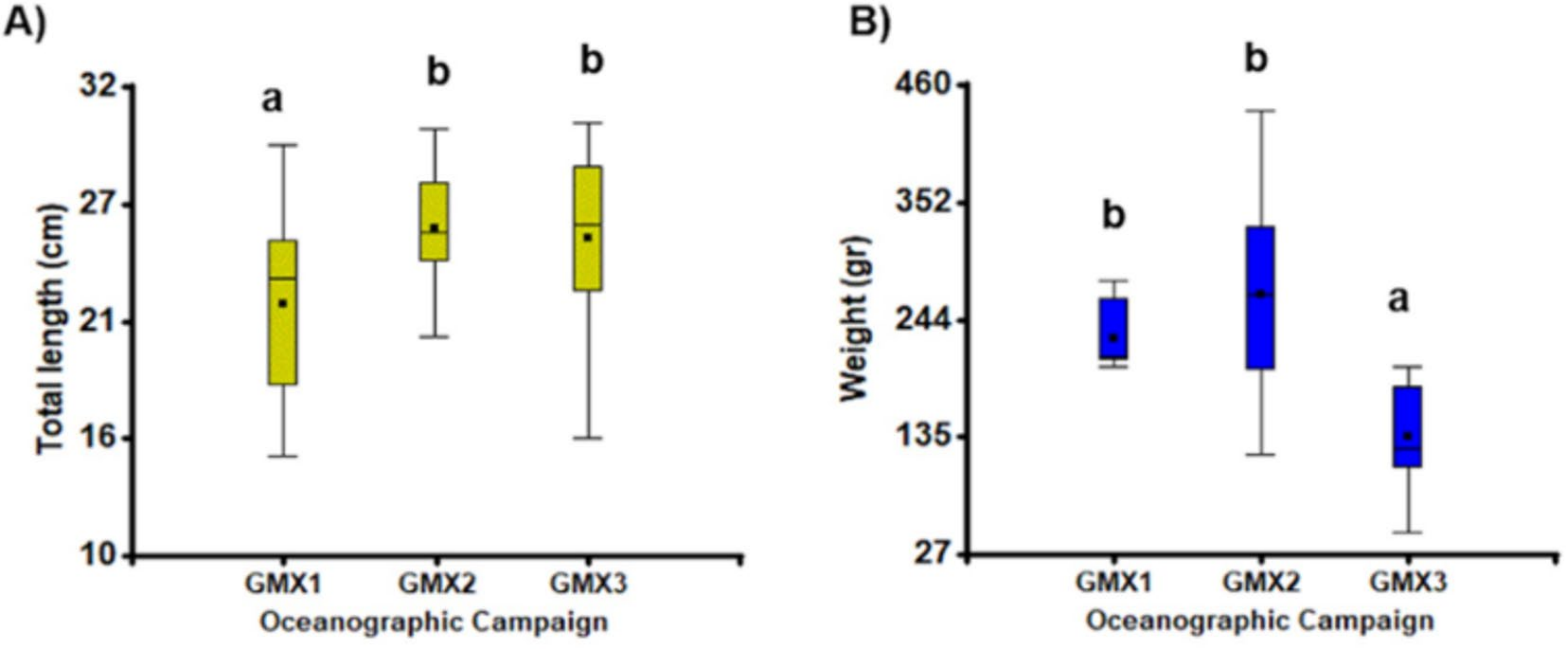
Statistical differences in the length and weight of all flatfish for three Gomex (GMX) oceanographic cruises. **A** Mean total length ± S.D. **B** Total weight average ± S.D. Different letters denote significant differences (*p* < 0.05). We used the Kruskal–Wallis nonparametric one-way ANOVA test

A total of 153 adult flatfish (*Syacium papillosum*) individuals were captured from 15 sites in 2010, 19 sites in 2011, and 17 sites in 2012 out of 87 sampled sites per year. Fish were captured between 15 and 200 m deep (Fig. 1B and Supplementary material Table S1). Most fish collected during Gomex-1 came from sites at 100 m depth at the eastern and western boundaries of the Yucatan shelf (Supplementary material Table S1: transects A and P, respectively). For Gomex-2 and Gomex-3, it was also possible to collect fish from the F–M transects at 50 to 200 m depth (Fig. 1). The size and weight of collected fishes varied between 20 and 28 cm of total length and from 105 to 270 g of weight. The total length of fishes in Gomex-2 and Gomex-3 presented significant differences compared to Gomex-1, and the weight of the fishes from Gomex-1 and Gomex-2 had significant differences to Gomex-3 (*H* = 6.97; df = 2; *p* = 0.0307 and H = 27.92; df = 2; *p* = 0.0001 to length and weight, respectively) (Fig. 3; Supplementary material Table S1).

### Histopathological findings

The external fish examination did not show clinical signs of diseases, injuries, or malformations. Some of the lesions observed were caused by mechanical damage during trawling. Between 8 and 14 types of histopathological alterations were identified in the selected organs (Supplementary material Table S2). The spleen was the organ with the fewest histological lesions, and the liver and kidney were the tissues that showed the most significant histopathologies by number and severity. The lesions prevalence values varied between 3% (gills hypertrophy) and 100% of melanomacrophage centers (MMCs) in the spleen, kidney, and liver. The prevalence values of each lesion were variable among cruises. There was a general reduction of lesion prevalence for the second year concerning to the first, increasing again for the third year (Supplementary material Table S2). However, the histopathologies were focal in most cases.

**Fig. 4:**
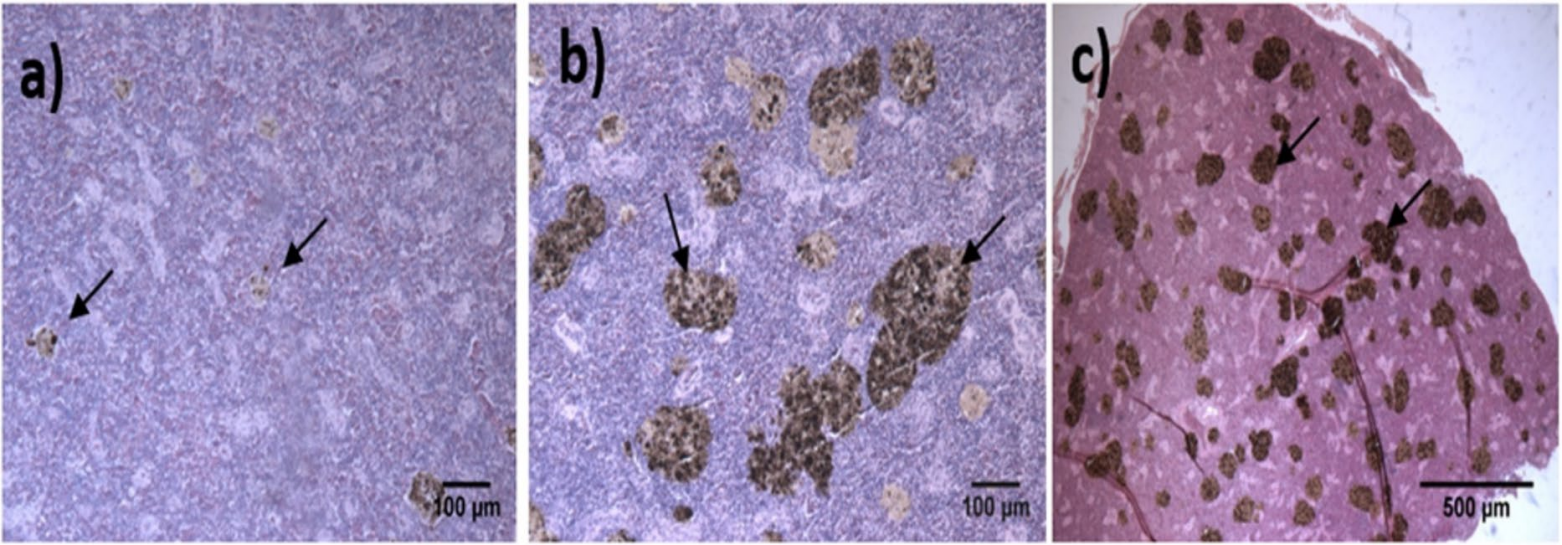
Histological lesions in spleen tissue during the Gomex 1, 2, and 3 cruises, **a** normal tissue with focal melanomacrophage centers (↑), 10 × ; **b**, **c** splenic tissue with abundant melanomacrophage centers (↑), 10 × and 4 × , respectively. H&E staining

The spleen presented eight lesions; however, the most prevalent alteration in the three cruises was the presence of MMCs, which in definition contain a varying amount of pigments within vacuoles, these pigments can include both classical melanin, including hemosiderin, and lipofuscins (Agius and Roberts, 2003) (Supplementary material Table S2; Fig. 4). This histological response reached a prevalence higher than 80%, which was maintained for all three OCs.

**Fig. 5:**
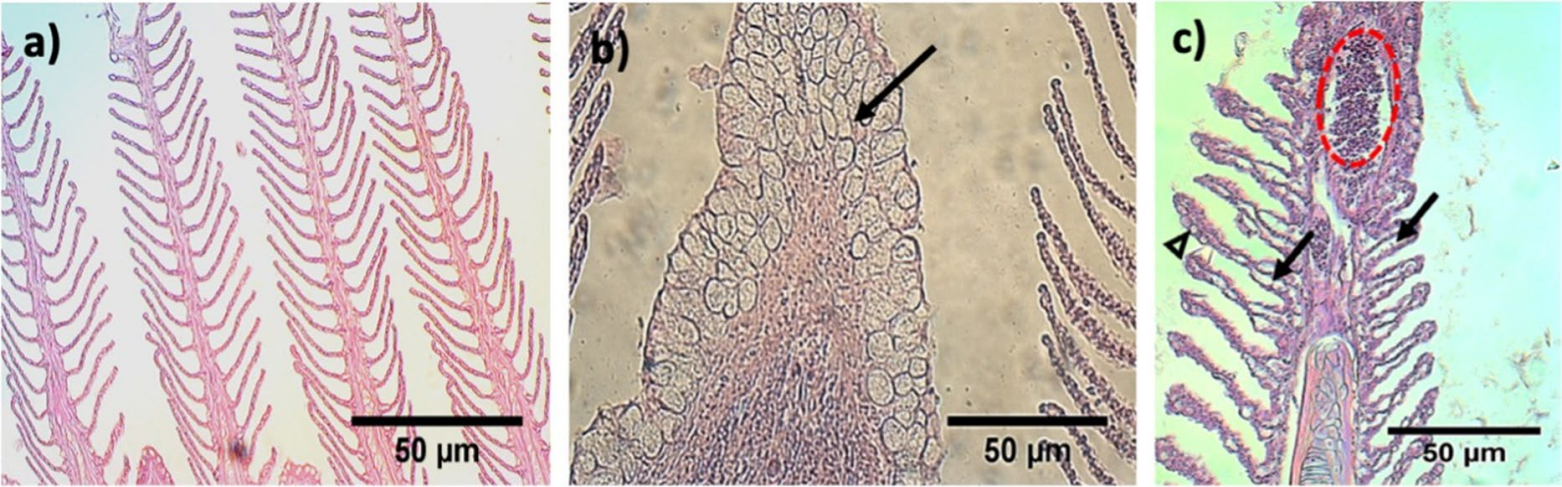
Histological lesions in gills of flatfish *Syacium papillosum* during oceanographic cruises Gomex 1, 2, and 3. **a** Healthy gill tissue, 10 × ; **b** increase in mucus at the branchial apex (metaplasia) (↑), 20 × ; **c** extensive epithelial lifting in gills with severe lamellar edema (↑), blood congestion (red oval), and mucus cells (Δ), 20 × . H&E staining

Thirteen histological damages were identified in the gills (Supplementary material Table S2). The prevalence of hypertrophy was 90% in samples from Gomex-1, while in Gomex-2 and Gomex-3, the values decreased to 20% and 3%, respectively. The prevalence of hyperplasia for the three cruises presented values > 89%. The prevalence of inflammation reflected in mucus cells and lamellar fusion tended to decrease through each OCs from 90 to 40%. At the same time, telangiectasia increased its prevalence through every OC (Supplementary material Table S2). The lamellae presented edema, metaplasia, and parasites (mainly protozoa), with 35 to 61% prevalence. The pathological alterations caused by the presence of parasites were usually limited to slight inflammation around the parasite cyst (Fig. 5).

**Fig. 6:**
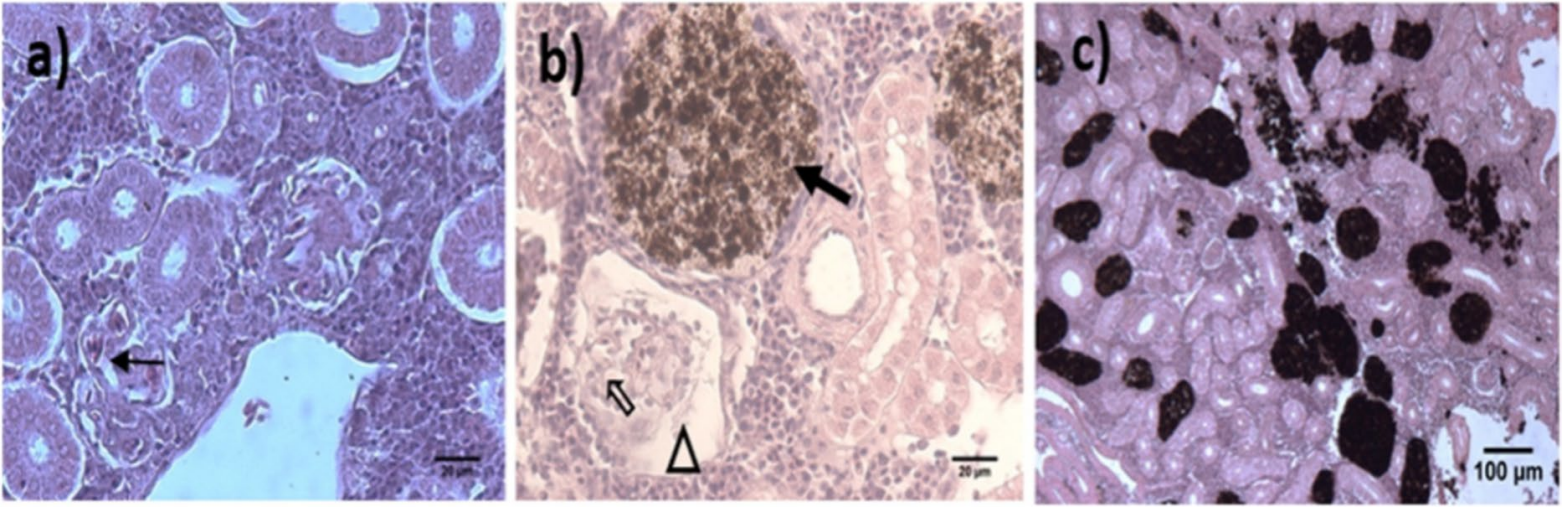
Histological lesions observed in kidney during the Gomex 1, 2, and 3 cruises. **a** Renal tissue with severe dilation in the glomerular vessels (↑), 40 × ; **b** renal tissue with abundant MMCs (  ), glomerular atrophy (Δ), and dilation of glomerular blood vessels (  ), 40 × ; **c** renal tissue with abundant MMCs, 10 × . H&E staining

MMCs were highly prevalent in the kidneys in each subsequent oceanographic cruise, with 85, 95, and 100%, respectively. However, cellular atrophy and necrosis were the most severe lesions, and they tended to increase through every OC (Supplementary material Table S2; Fig. 6). The rest of the pathologies observed in this organ varied in percentage on all three cruises.

**Fig. 7:**
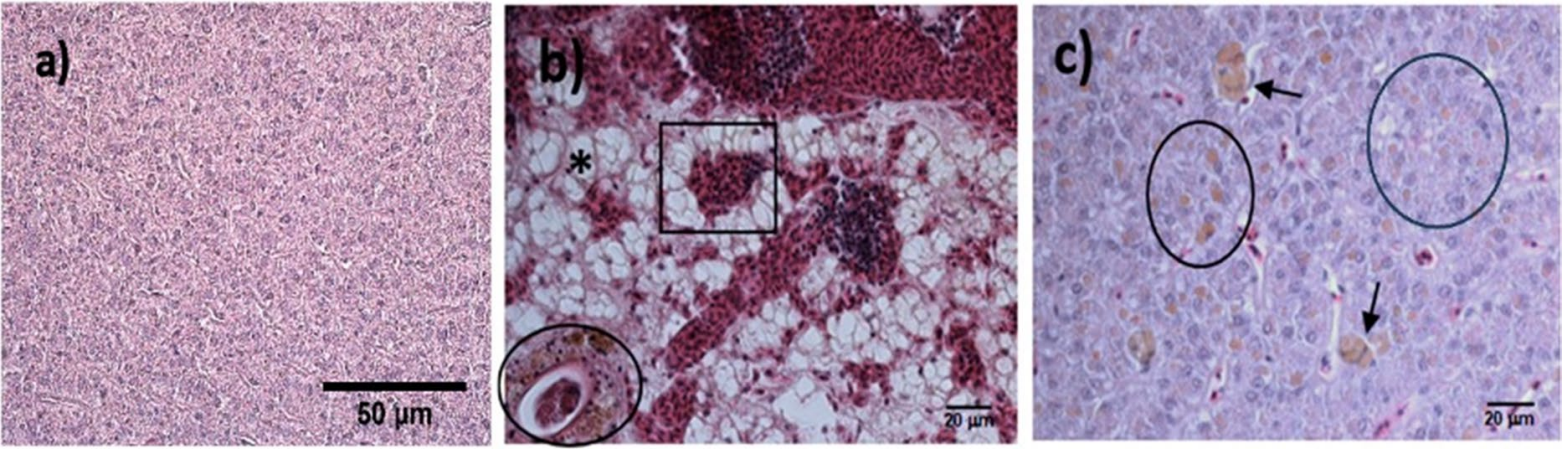
Histological lesions observed in the liver during the Gomex 1, 2, and 3 cruises. **a** Normal liver tissue, 40 × ; **b** congestion of hepatic sinusoids (□), MMCs (○), severe fatty degeneration (*), 40 × ; **c** liver tissue with abundant MMCs (↑) and extended hyaline droplets (black circle), 40 × . H&E staining

In the hepatic tissue, we identified 13 lesions where cellular atrophy, fatty degeneration, MMCs, and necrosis were the most prevalent (> 80%) (Supplementary material Table S2, Fig. 7). Additionally, there was an increase in the prevalence of these lesions through the OCs. Injuries such as inflammation, hemorrhage, granulomas, dilation of sinusoids, adenomas, and parasites showed lower prevalence (< 50%). Again, the injuries caused by parasites were focal (slight inflammation).

The histological damage, like focal inflammation in the kidney, liver, and spleen and focal telangiectasia in the gills, was slight on most sampling sites. However, in two sampling sites of Gomex-2 (G34 and H40) and Gomex-3 (G35 and H40) (Fig. 1B), the fishes showed severe histological damage such as fatty degeneration in the liver accompanied by cellular atrophy, necrosis, and hypertrophy in the renal epithelial cells, as well as abundant MMCs in the kidney and spleen.

### Quantification of histological damage in *S. papillosum*

Table 1 shows the classification of the histopathological alterations. Table 2 gives the mean values of the extent of the damage (EOD) and histological alteration index (HAI) in organs and tissues for the different lesions observed in the liver, spleen, kidney, and gills of flatfish collected from the Yucatan shelf during the Gomex cruises. The extent of the damage (EOD) of the pathological alterations in the evaluated organs was mainly focal (Grade 2) and generally classified in DTC stage II, except for the neoplastic lesion, severe necrosis, and structural disintegration, which were classified as DTC stage III (Supplementary material Table S2). The highest values of HAI in the liver and kidney are from the coastal zone of the Yucatan shelf, mainly off the coast of Progreso and in front of the upwelling zone, close to the Mexican-Caribbean coast.

**Table 2:** Mean and standard deviation of the extent of damage (EOD) and histological alteration index (HAI) in the liver, spleen, kidney, and gills of the flatfish *Syacium papillosum* from the continental shelf of the Yucatan Peninsula. These data were obtained during oceanographic cruises Gomex 1, 2, and 3

TISSUE		GOMEX 1 Mean/Std.Dev	GOMEX 2 Mean/Std.Dev	GOMEX 3 Mean/Std.Dev
LIVER	EOD	1.34 ± 0.27 a	1.29 ± 0.29 a	1.37 ± 0.12 a
SPLEEN	EOD	1.21 ± 0.36 b	1.13 ± 0.37 a	1.28 ± 0.29 b
KIDNEY	EOD	1.28 ± 0.46 b	1.25 ± 0.28 a	1.41 ± 0.18 b
GILLS	EOD	1.56 ± 0.49 c	1.34 ± 0.22 b	1.27 ± 0.23 a
LIVER	HAI	131 ± 93 c	108 ± 59 c	35 ± 40 b
SPLEEN	HAI	2 ± 3 b	3 ± 4 a	5 ± 7 b
KIDNEY	HAI	97 ± 70 c	58 ± 60 b	19 ± 18 b
GILLS	HAI	30 ± 19 c	7 ± 6 b	4 ± 3 a

Different letters denote significant differences between values (*P <0.05*)

**Fig. 8:**
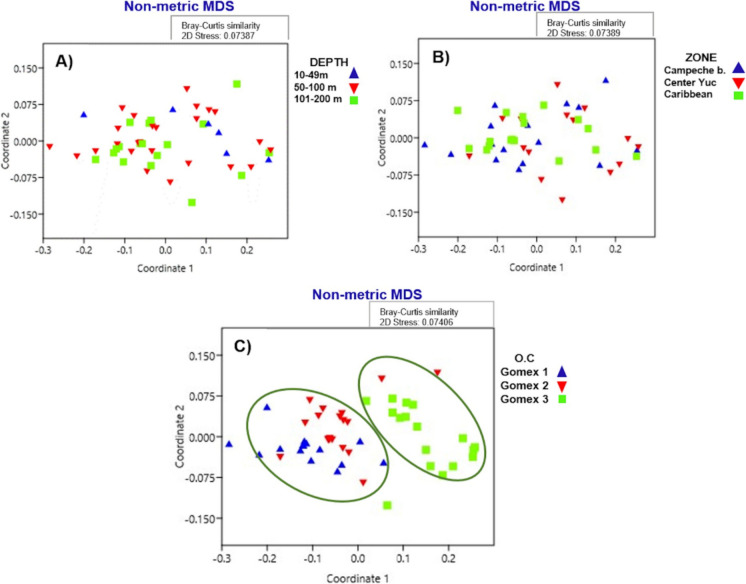
Nonmetric multidimensional scaling (NMDS) of the histological alteration index (HAI) of the dusky flounder *Syacium papillosum*. **A** NMDS analysis between HAI values with the depth classification of the Gomex 1, 2, and 3 cruises. **B** NMDS analysis between HAI values with zone classification of the Gomex 1, 2, and 3 cruises. **C** NMDS analysis between HAI values and years of Gomex cruises within the Yucatan platform

The NMDS analyses showed that, although the HAI values of some fish collected in different zones of the Yucatan shelf were high (> 100), they were not associated with depth or sampling zone (Fig. 8A, B). In contrast, Fig. 8C shows differences among oceanographic cruises.

### RDA between HAI and abiotic variables: contaminants, physicochemical parameters, and nutrients

**Fig. 9:**
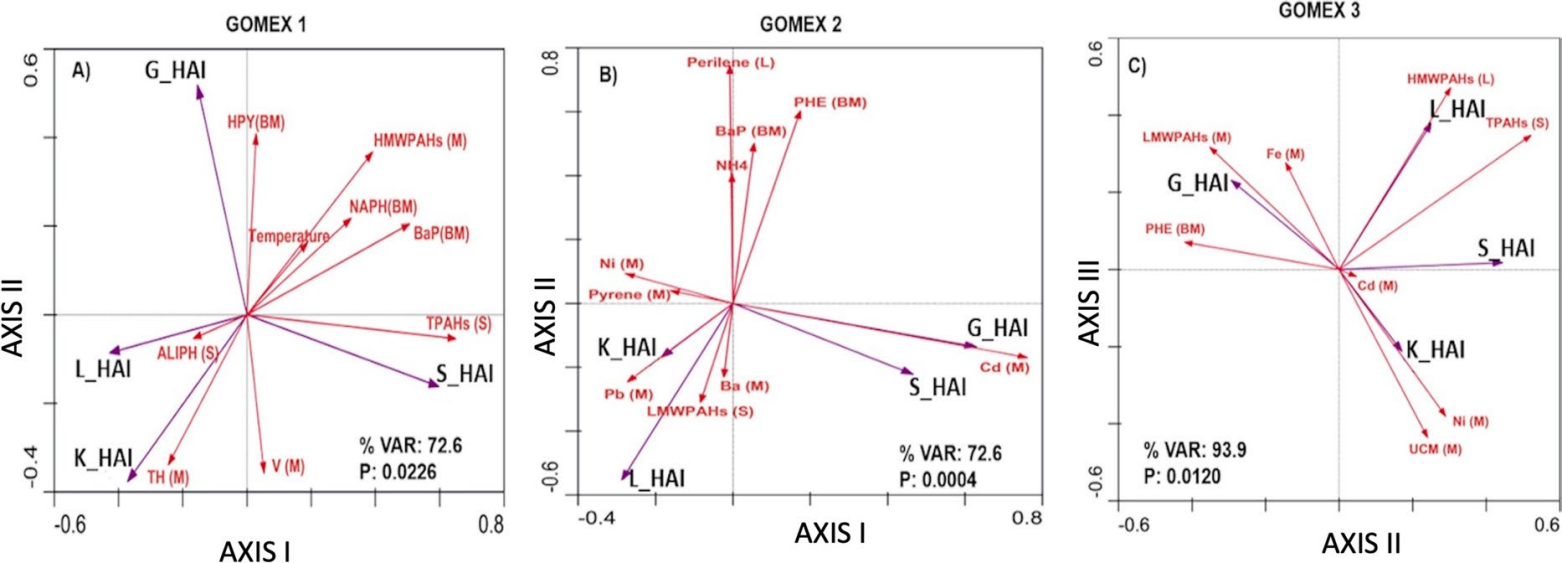
**A**–**C** Redundancy analyses (RDA) of the environmental variables and histological alteration index HAI of *Syacium papillosum* of the three Gomex cruises (1, 2010; 2, 2011; and 3, 2012). **A** For Gomex-1, the RDA accounted for 72.6% of the total variance and was highly significant for the first and all canonical axes (*F* = 6.3473; *p* value = 0.0226; 4999 permutations). **B** For Gomex-2, the RDA accounted for 72.6% of the total variance and was highly significant for the first and all four canonical axes (*F* = 9.928; *p* value = 0.0004; 4999 permutations). **C** For Gomex-3, the RDA accounted for 93.9% of the total variance and was highly significant for all four canonical axes (*F* = 2.307; *p* value = 0.0120; 4999 permutations). Abbreviations: L_HAI, liver-histological alteration index; S_HAI, spleen-histological alteration index; K_HAI, kidney-histological alteration index; G_HAI, gill-histological alteration index; TH (M), total hydrocarbons (muscle); ALIPH (S), aliphatic hydrocarbons (sediment); TPAHs (S), total polycyclic aromatic hydrocarbons-sediment; BaP(BM), benzo[a]pyrene (bile metabolites); HPY (BM), hydroxypyrene (bile metabolites); NAPH (BM), naphthalene (bile metabolites); HMWPAHs (M), polycyclic aromatic hydrocarbons–high molecular weight (muscle); PHE (BM), phenanthrene (bile metabolites); Ni (M), nickel (muscle); Ba (M), barium (muscle); Cd (M), cadmium (muscle); Pb (M), lead (muscle); V (M), vanadium (muscle); Fe (M), iron (muscle); Pyrene (M), pyrene (muscle); Perylene (L), perylene (liver); LMWPAHs (S), polycyclic aromatic hydrocarbons–low molecular weight (sediment); NH4, ammonia; UCM (M), unresolved complex mixture (muscle); LMWPAHs (M), polycyclic aromatic hydrocarbons–low molecular weight (muscle); HMWPAHs (L), polycyclic aromatic hydrocarbons–high molecular weight (liver)

The redundancy analyses (RDA) showed significant and positive associations between the HAI values of the liver, kidney, spleen, and gills (dependent variables) and independent variables such as polycyclic aromatic hydrocarbons (PAHs) and heavy metal concentrations. Although other variables, such as nutrients, water physicochemical variables, fish length, weight, and *K* as a covariables, were considered, these were not positively associated with the histological index. For Gomex-1, the RDA accounted for 72.6% of the total variance and was highly significant for the first and all canonical axes (*F* = 6.3473; *p* value = 0.0226; 4999 permutations). The HAI of the liver and kidney (L_HAI and K_HAI) were positively associated with the total hydrocarbons in muscle (TH (M)) and with aliphatic hydrocarbons in sediments (ALIPH (S)); in contrast, the spleen HAI values (S_HAI) were positively associated with total polycyclic aromatic hydrocarbons in sediment (TPAHs (S)) and with vanadium in muscle (V (M)). For gill HAI (G_HAI), there was a close association with hydroxypyrene bile metabolites (HPY (BM)) (Fig. 9A). For Gomex-2, the RDA accounted for 72.6% of the total variance and was highly significant for the first and all four canonical axes (*F* = 9.928; *p* value = 0.0004; 4999 permutations). The HAI for kidney and liver (L_HAI and K_HAI) were positively associated with PAHs of low molecular weight in sediment (LMWPAHs (S)), lead, and barium in muscle (Pb (M) and Ba (M)), while the spleen and gills’ HAI (S_HAI and G_HAI) were positively associated with cadmium in muscle (Cd (M)) (Fig. 9B). For Gomex-3, the RDA accounted for 93.9% of the total variance and was highly significant for second and third canonical axes (*F* = 2.307; *p* value = 0.0120; 4999 permutations). The HAI of the liver and spleen (L_HAI and S_HAI) were positively associated with PAHs of high molecular weight (HMWPAHs (L)) and total PAHs in sediment (TPAHs (S)). The kidney HAI (K_HAI) was positively associated with the UCM and nickel in muscle (UCM (M) and Ni (M)), while the gill HAI (G_HAI) was closely associated with low molecular weight PAHs (PAHs LMW (M)), phenanthrene (PHE (BM)), and iron in muscle (Fe (M)) (Fig. 9C).

## Discussion

Our original hypothesis that severe histological lesions would be present in *S. papillosum* at the closest sites to the DWH oil spill, and therefore at the boundary of the Yucatan shelf, was not fulfilled. The distribution of lesions in *S. papillosum* from the Yucatan shelf showed that these occurred at all sampling sites, being more prevalent and severe in the eastern and western margins of the YS and in the coastal zone in front of Progreso harbor. Regarding the severity of the lesions, the DTC of the collected fish from all Gomex oceanographic cruises showed severe alterations in the liver and kidney (stage III), which can compromise the normal function of these organs. The observed histological damage could be the consequence of chronic exposure to hydrocarbons and heavy metals present in the sediments (Costa et al., 2009; Yancheva et al., 2016) (Fig. 9). Vidal-Martínez et al. (2019) mention that although there are no oil extraction activities in the YS, a possible explanation for the presence of pollutants causing *S. papillosum* injuries is the proximity of the sampling sites to the oil extraction operations of the Campeche Bank in the western YS and to the loop current carrying these pollutants into the GoM from the Caribbean area in the eastern YS. Moreover, Peters et al. (2021) mention that the eddies formed by the loop current create strong currents that redistribute the water in the surface layers, transporting the pollutants mainly on continental shelves and along the coastal zones of GoM. In addition, according to Love et al. (2013), the study area matches spatially with the presence of hydrocarbon seeps in the Yucatán Peninsula, which could be related to the most severe histological injuries and highest HAI scores in *S. papillosum*.

Concerning the severity of pathological changes (DTC), most alterations did not hurt the organ structure and function of the liver. Fatty degeneration, MMCs, and necrosis increased their prevalence in fishes of Gomex-2 and Gomex-3. The presence of lesions such as necrosis in liver tissue is focal and does not represent an irreversible change in the liver tissues. These lesions have been associated with biochemical disturbances such as degenerative alterations (granular, vacuolar, hydropic, and fatty degeneration) (Monteiro et al., 2005; Rajeshkumar & Munuswamy, 2011). In addition, we occasionally found foci of cellular alteration (FCA), structural disintegration, and coagulative necrosis, which are pathologies that remain and can extend despite the disappearance of the causal factor. These lesions have been reported previously and linked to PAH exposure (Myers et al., 2003). The extension of these lesions can often affect liver functions, such as the detoxification process and fat metabolism, carbohydrates, and proteins (Straif et al., 2005).

The severity of the histological damages in sampling sites near the coastal area of Yucatan (B6) may be due to strong freshwater discharge from the continent during the rainy season, which contains different compounds, including nutrients such as HCO3, SO4, Cl, Ca, Mg, Na, K, NO2, NO3, and NH4 (Delgado et al., 2010; Herrera-Silveira & Morales-Ojeda, 2010). Additionally, there is evidence of low and high molecular weight PAHs in sediments all along the coastal lagoons of Yucatán, presumably carried from the continent to the coastal region by subterranean currents (Gold-Bouchot et al., 2014). Consequently, fish are exposed to all these compounds in the coastal zone, which could produce the histopathologies found in *S. papillosum.* On the other hand, histopathological damages observed in the deepest sampling stations (G34, G35, and H40) could be related to additional factors such as pollutants dispersion and frequent maritime traffic (including oil transportation), which often results in frequent small-scale spills, that is one of the main stressors in approximately 50% of the YS (Ocaña et al., 2019).

Furthermore, the RDA analyses suggested associations or causal relationships between the concentrations of hydrocarbons, heavy metals, and the HAI scores of *S. papillosum*. Despite the positive association between histopathological lesions and the presence of pollutants shown by our analyses, we should not jump to conclusions regarding the source of such pollutants being from the oil industry only. As Vidal-Martínez et al. (2019) point out, the concomitant effect of other environmental variables and other water sources of the discharge into the YS should be considered. To develop a histological lesion, high pollutant concentrations are not always necessary, and low doses are usually sufficient to trigger their development (Améndola-Pimenta et al., 2020). This indicates that localized environmental conditions could lead to similar fish histopathologies elsewhere on the GoM.

In the year of the Deepwater Horizon oil spill, we observed some grade of histological lesions in all fishes from Yucatán shelf stations sampled. However, some sampling stations assessed in A and B transects at western YS where there are not oil activities and are distant from posible crude oil spills can serve as a kind of “baseline/reference,” because they showed minimal or low concentrations of pollutants, such as total polycyclic hydrocarbons, total hydrocarbons, their metabolites, and heavy metals (Supplementary material S1). Although no solid evidence was found of DWH pollutants reaching the YS, the decreasing tendency in prevalence and severity of lesions found in *S. papillosum* throughout the Gomex cruises is similar to the pattern observed by Murawski et al. (2014). These authors found a similar pattern while sampling considerably near the DWH oil spill. The incidence of skin lesions reported in their 2011 samplings had decreased by 53% when they sampled again by 2012, with the severity of lesions also declining. A likely reason for the decreased prevalence and severity of lesions in Murawski’s research and our own could be the death of the more severely affected fish. It is not yet reasonable to conclude that the histopathologies reported in this paper have been necessarily caused by pollutants originated from the DWH or that the observed decrease in lesion prevalence observed by Murawski and ourselves can be indeed attributed to the death of the most severely affected fish. More precise conclusions on these matters will require additional long-term research.

## Conclusions

The prevalence and high HAI values found in the examined organs of flatfish inhabiting the Yucatan platform are evidence of specific areas with poor environmental quality. The histopathologies described in this work also indicate that these fish are chronically exposed to stress sources.

These findings constitute a relevant baseline data set for monitoring the pollutants and their biological effects in the YS. The Yucatan shelf has several anthropogenic and natural stress factors, thus requiring continuous biological and environmental monitoring to understand their effect on the YS communities. Equally important will be experimentally testing the potential relationship between contaminants such as hydrocarbons and heavy metals with histological damage in *S. papillosum*.

## Supplementary Information

Below is the link to the electronic supplementary material.

ESM 1 (XLSX 48.1 KB)

## Data Availability

No extra datasets were generated or analysed during the current study.
